# Considerations for Assessing Maximum Critical Temperatures in Small Ectothermic Animals: Insights from Leaf-Cutting Ants

**DOI:** 10.1371/journal.pone.0032083

**Published:** 2012-02-24

**Authors:** Pedro Leite Ribeiro, Agustín Camacho, Carlos Arturo Navas

**Affiliations:** Departamento de Fisiologia, Instituto de Biociências, Universidade de São Paulo, São Paulo, Brazil; University of Arizona, United States of America

## Abstract

The thermal limits of individual animals were originally proposed as a link between animal physiology and thermal ecology. Although this link is valid in theory, the evaluation of physiological tolerances involves some problems that are the focus of this study. One rationale was that heating rates shall influence upper critical limits, so that ecological thermal limits need to consider experimental heating rates. In addition, if thermal limits are not surpassed in experiments, subsequent tests of the same individual should yield similar results or produce evidence of hardening. Finally, several non-controlled variables such as time under experimental conditions and procedures may affect results. To analyze these issues we conducted an integrative study of upper critical temperatures in a single species, the ant *Atta sexdens rubropiosa*, an animal model providing large numbers of individuals of diverse sizes but similar genetic makeup. Our specific aims were to test the 1) influence of heating rates in the experimental evaluation of upper critical temperature, 2) assumptions of absence of physical damage and reproducibility, and 3) sources of variance often overlooked in the thermal-limits literature; and 4) to introduce some experimental approaches that may help researchers to separate physiological and methodological issues. The upper thermal limits were influenced by both heating rates and body mass. In the latter case, the effect was physiological rather than methodological. The critical temperature decreased during subsequent tests performed on the same individual ants, even one week after the initial test. Accordingly, upper thermal limits may have been overestimated by our (and typical) protocols. Heating rates, body mass, procedures independent of temperature and other variables may affect the estimation of upper critical temperatures. Therefore, based on our data, we offer suggestions to enhance the quality of measurements, and offer recommendations to authors aiming to compile and analyze databases from the literature.

## Introduction

Climate warming has stimulated integrative studies aiming to assess or predict the impact of environmental temperatures on faunas. This complex problem has been addressed from several perspectives, one of which is how thermal climatic events affect individual performance and, in turn, may cause population declines [1], [2]. Most such studies have focused on various views of the thermal tolerances of ectothermic animals because they represent the vast majority of animal species and many are known to have thermally dependent behavioral and physiological functions. Therefore, a main tenet is that measures of thermal constraints, studied in parallel with thermal preferences and environmental temperatures, provide information about the vulnerability of organisms to changing temperatures [3]–[25]. From this perspective, vulnerability (an inference about ecological performance) may be expressed as a correlate of the difference between the expected body temperature in a given scenario (an autoecological parameter) and indicators of maximal thermal tolerance (a physiological parameter) [6], [26]. Relying on diverse indicators of thermal limits (see definitions in [27], Table 3.4 and [1], Table 1), this approach to assess vulnerability has been applied to several contexts and systematic groups [28]–[35]. However, if heating rates influence upper thermal limits, these limits may differ among species when assessed at a given heating rate, yet be similar when ecologically relevant heating rates are considered for each species. Therefore, analyses of vulnerability based on upper thermal limits, and general considerations regarding ecological implications of critical temperatures, require considerations about heating rates [1]. In addition experimentally determined thermal limits rely on two major (but usually tacit) assumptions. One assumption is that these limiting values reflect the maximum temperature tolerated by animals before they suffer permanent physiological damage (because damage would indicate that these limits were actually surpassed in the test and therefore the estimate was more a lethal rather than a critical upper limit). Another assumption is that thermal limits are reproducible traits of individuals, so that the average values from several individuals define the thermal tolerances of a population. However, these major assumptions require experimental validation. Finally, hidden and uncontrolled sources of variation, e.g., protocols (time of the day, end point and others) and experimental procedure (time under test conditions, manipulation and others) may add complications to the interpretation of data through unplanned effects on physiology. The aims of this paper are to test in a single experimental model the 1) influence of heating rates in the experimental evaluation of upper critical temperature, 2) assumptions of absence of physical damage and reproducibility, and 3) sources of variance often overlooked in the thermal-limits literature; and 4) to introduce some experimental approaches that may help researchers to separate physiological and methodological issues.

**Table 1:** Heating rates treatments and sample size.

Group	Heating rate	Number of ants that recovered after 2 hours of CTMax test
1	2°C/1 min	59
2	1°C/1 min	30
3	0.66°C/1 min	30
4	0.5°C/1 min	30
5	0.4°C/1 min	30
6	0.33°C/1 min	30
7	0.29°C/1 min	30
8	0.25°C/1 min	37
9	0.22°C/1 min	31
10	0.2°C/1 min	39
11	0.18°C/1 min	33
12	0.16°C/1 min	30

The thermal tolerance of animals is a plastic, environmentally-induced trait that responds to planned experimental sources of variance [18], [36]–[45], including past thermal history [37], acclimation [36], [39], [40] and acclimatization [46]. It also varies with ontogenetic stage [47] and with seasonal and daily biological cycles [48]–[50]. These known sources of variation need to be recognized if thermal tolerances are calculated. Thus, researchers must control or standardize measures in terms of ontogeny, reproductive state, season and thermal history. However, less obvious sources of variance may enhance intra-specific variation and produce a failure to detect differences among species (increase Type II error), or may exaggerate the relevance of minor differences. Critical temperatures may be influenced by the mass of tested individuals [51], the heating rates applied [18], [51]–[54] and indicators of experimental endpoint such as lack of response, muscular spasms [55], or thermolimit respirometry [56]. In addition, the analysis of critical temperatures may be based on ramping or static methods (e.g. knock-down critical temperatures (see [57]). Given this methodological diversity, both uncontrolled sources of variation in physiological states and methodological issues may affect the determination of thermal limits in ectothermic animals. However, several important sources of variation have been studied largely independently and in different taxa and a single-species integrative analyses are much needed.

We chose ants of the same colony as an ideal model in our study, because large numbers of individuals are available, remarkable differences in body size exist among individuals possessing similar genetic makeup, and colonies can be maintained in captivity under controlled conditions. To assess upper critical temperatures, we followed classical approaches for studying the parameter known as the critical thermal maximum (CTMax). The CTMax figures among the first parameters proposed to link animal thermal physiology and ecology [58]. It has been widely used to investigate thermal limits in ectothermic animals [59] and has been applied in the context of climate warming [16], [24], [60]–[62]. We investigated the effects of heating rates on the CTMax, and assess damage and reproducibility of thermal limits by focusing on whether tests had an impact on subsequent measures. If an impact occurred, we sought to determine whether usually overlooked sources of variance (duration of experiments, procedures other than thermal treatment, daily rhythms and body mass) could be responsible for the observed variation in the CTMax. For example, the duration CTMax experiments is inevitably correlated with heating rates and with the magnitude of the temperature turning out to be the CTMax for a given species (e.g., if heating rates are constant, greater CTMax require longer experiments). Therefore, researchers must know whether effects on CTMax are truly derived from heating rates themselves or from collateral effects of extended manipulation. Daily rhythms and mass-dependent responses were additional concerns addressed by the study. We also present detailed protocols and analyses that shall help researchers to tell apart effects of some sources of variation. Our results generated a series of recommendations that contribute to current debate and are useful for investigations of organismal upper thermal limits, independently of the method used.

## Results

**Figure 1:**
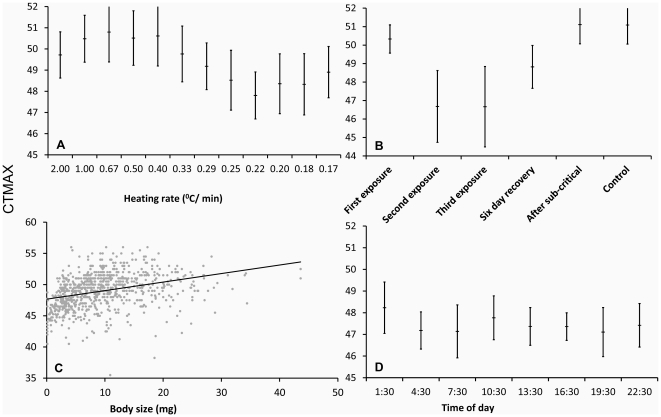
Results of CTMaxs tests. a) CTMaxs measured at different heating rates from 2°C/min to 0.16°C/min. b) CTMax estimates corresponding to different contrasts: i) the contrast between the first exposure*, and second and third exposures, to determine reproducibility under short-time recovery (each exposure with a 24 h interval); ii) the contrast between the first exposure* and the six-day recovery, to determine reproducibility under long-time recovery (144 h); iii) The contrast between the first exposure* and the CTMax after subcritical exposure and the manipulation control. c) Correlation between body mass and CTMax. d) CTMax measured at different times of the day. Bars indicate SDs. *The results from the first days of the short- and long-time recovery experiments are plotted together.

Heating rates determined final CTMax readings. Within the range of heating rates explored (2°C/min to 0.16°C/min [1°C per 6 min]), faster heating led to a higher CTMax (ANCOVA: F_3,400_ = 35.5695, p<0.001, Figure 1a). This effect was due to heating rates *per se*, and not to extended experiments as a byproduct of slower heating rate (see results on a Procedure-control below).

Our test of reproducibility compared the CTMax of marked individuals measured more than once, always recovering under normal colony life between tests. These tests indicated that CTMax values are not reproducible in *Atta sexdens rubropilosa*. Exposure to a first measure of CTMax caused a decrease in two subsequent CTMax readings, one performed 24 h after the first measure and a third one 24 h after the second one (ANCOVA: F_2,40_ = 8.5104e+25, p<0.001, Figure 1b). However, the effect was not cumulative, and second and third CTMax measures were comparable (Tukey test: p = 0.985). An additional test of reproducibility of CTMax was made six days after a first measure. This test also led to reduced CTMax, although the observed reduction was lower in magnitude than those observed in reproducibility tests after 24 h and 48 h (ANCOVA: F_3,71_ = 17.745, p<0.001, Figure 1b). The above results seem derived from exposure to critical temperatures and not from responses of tested individuals to any other aspect of the experiment. This is so because a sub-critical temperature control test (temperatures elevated to high, yet subcritical values, see materials and methods) did not alter subsequent CTMax readings after 48 h (ANCOVA: F_3,46_ = 0.0012, p = 0.973, Figure 1b). Similarly, CTMax tests controlling for experimental procedures other than thermal treatment, and focusing mainly on the period of time under experimental manipulation (see procedure-control test in Material and Methods) produced values of CTMax similar to those performed in the absence of such control. Procedure-control tests generated CTMax data comparable to that generated in the first CTMax performed (ANCOVA: F_3,99_ = 1.338, p = 0.250) and the test exploring long-time recovery (ANCOVA: F_3,82_ = 1.296, p = 0.258, Figure 1b). In summary, we observed reduced reproducibility of CTMax only in tests that actually exposed individuals to upper critical temperatures, not in any control test comparable regarding manipulation and procedure, but that did not exposed animals to CTMax values.

Daily cycles did not influence the CTMax (ANCOVA: F_3,223_ = 1.464, p = 0.162, Figure 1d). In contrast, ant body mass affected upper thermal tolerances. Larger individuals ants tolerated higher temperatures than smaller (linear regression R^2^: F_1,839_ = 187.6, R = 0.182, p<0.001, Figure 1c). The interaction between body mass and heating rate was not significant (ANCOVA: F_3,400_ = 0.032, p = 0.954), as would have been expected if thermal inertia were an issue.

## Discussion

Our data confirm that heating rates are influential in the estimates of critical temperatures [51]–[54], [63]. The trends found in *Atta sexdens rubropilosa* are likely general for the species (not necessarily in absolute values, which may vary among colonies [3], but on main patterns and correlations. In addition, these trends are generally compatible with those presented by Rezende et al. [18], but we do not postulate any specific mechanism as a causal agent of the pattern observed. Our argument is that thermal increase in ectothermic animals will produce two simultaneous phenomena. First, both exposure to temperature and manipulation may affect the physiological performance of test animals for diverse reasons, among them the explanations hypothesized by Rezende et al. [18]. From this viewpoint, it is probable that longer experiments will reduce the health of animals under experimental conditions and will reduce tolerance (through mechanisms including but not limited to desiccation, loss of energy reserves and oxygen-limited thermal tolerance; see Portner et al. [64], Peck et al. [54] and Rezende et al. [18]). Oxygen-limited thermal tolerance may have influenced the trends found in *Atta sexdens rubropilosa*, because the observed reduction in thermal tolerance was related only to exposure to upper critical temperatures, and not to any other aspect of experimental manipulation. On the other hand, exposure to increasingly high temperatures may activate physiological responses that enhance thermal tolerance and that have different temporal courses. Thus, trends enhancing or decreasing thermal tolerance are possible, even more as experiments become longer (i.e., as heating rates become slower or CTMax values turn out to be higher). Accordingly, the dominant trend for a given group of experimental animals will depend on both taxon-specific and experiment-specific considerations. Under this model, it would not be possible to anticipate the impact of heating rates in a new organism to be tested. However, given nuances of individual variation, slow heating rates leading to longer experiments may be associated with higher variances (as seen in Chown et al. [51]).

If heating-rate effects cannot be anticipated, authors targeting baseline thermal protection may prefer fast acute heating rates. In addition, fast heating rates are less likely to impose a lag in the homogenization of the body temperature, at least in small aquatic animals, such small fish [65]. However, the relationship between the heating power of the equipment, the size of the experimental animals, and the thermal conductivity of the media (e.g. air versus water) needs to be explored to guarantee uniform heating rates. This is more of a problem in aerial tests because the thermal conductivity of air is more than 20 times smaller than that of water. Although experiments can be fine-tuned in preliminary tests, some species of interest may provide only limited samples. In such cases, we suggests researchers opt for choose heating rates compatible with homogeneous heating in tested individuals.

A final important comment on heating rates addresses the dominant assumption that a higher baseline critical temperature (e.g., CTMax) confers greater thermal tolerances in the field [4], [27]. This very assumption supports the premise that safety ranges can be deduced from differences between critical temperatures and actual or predicted field temperatures. Support for this assumption emerges from the observation that species exposed to very high temperatures have particularly high critical temperatures [22], [62], [66]–[69] and, more recently, from broad meta-analysis [18], [70]. However, the current global scenario requires inference about ecological critical temperatures, that is, the thermal tolerance at typical (or predicted) rates of field temperature change [1]. Baseline and ecological critical temperatures are not necessarily the same, and we lack empirical studies generalizing the relationship between these two parameters (but see Rezende et al. [18], [70]). For example, two species differing in acute CTMax may exhibit similar values if compared at the heating rates typical of their microhabitat. This largely unexplored area requires additional data.

The key assumption of reproducibility of thermal limits has received minimal attention in the literature. A related assumption is that critical thermal limits refer to temperatures withstood by animals without suffering permanent damage [58], [59], a post-experimental state usually validated through simple behavioral observations. If these two assumptions are met, upper critical temperatures would be truly reproducible, generate similar values across tests, or eventually would indicate heat hardening [71], [72]. Alternative results have been observed in *Embioptera* [73] and in the juveniles of three species of arachnids, *Rhipicephalus sanguineus*, *Ixodes scapularis* and *Amblyomma americanum* [74]. Likely, hardening has variable temporal scales and vary according to thermal treatment, heating protocol and species. Independently of this consideration, our results suggest that critical temperatures are not necessarily reproducible among individuals, not because they are transient but because exposure to upper critical temperatures cause long lasting deleterious effects on the animals, even if recuperation is apparent through behavioral observations. Accordingly, the end point of the experiments as performed may have overestimated tolerances in our experiments, and overall, in the literature. This issue requires attention because it challenges the practice of evaluating CTMax recovery from simple post-test behavioral observations. The deleterious effects of a single CTMax tests were evident six days after that tests and reduced critical temperatures by more than 3°C. In addition, the variance in critical temperature was more than 7-fold higher in tests 2 (24 h after first tests) and 3 (48 h after first test) than in test one. This finding suggests that exposure to upper thermal limits affected individuals in different way, favors a simple dichotomic model (hardening versus health, see next paragraph), and poses straightforward practical implications: 1) even if recuperation is apparent, upper critical temperature experiments may overestimate tolerances; 2) simple behavioral tests are not ideal to demonstrate recuperation. Ideally, test should be repeated controlling for time in captivity and manipulation, but this may not be practical under many circumstances. Then, longer post-experimental observations and alternative species-specific options should be considered; and 3) if extrapolations are made from laboratory tests to the field, the frequency of near-critical events may affect ecological performance. One single event may be critical reducing tolerance to subsequent exposure.

Body mass is an important source of variance that is often unconsidered or not clearly associated with either physiological or methodological issues. Our results suggest that in *Atta sexdens rubropilosa* upper thermal limits are affected by body mass, and that this is not an artifact of mass-procedure interactions. The interaction between body mass and heating rate may be illustrative in this context: If this interaction explains variance in thermal limits, it is likely that experimental correlates of body size, such as heating rates or dehydration rates (e.g., Rezende et al. [18]) are complicating factors. In knock down experimental designs, heating rates may vary among individuals of different sizes, thereby increasing variance and reducing the power of the analysis. Although body size affects the CTMax of A. s. *rubropilosa*, these results cannot be obviously correlated with the ecology or behavior of ant castes. For example, we did not use the smallest ants, which are rarely exposed to high temperatures. So far studies in different species corroborate tendencies towards an increase [54] or decrease [75] of CTMax with body size; given the physiological, phylogenetic, methodological and scale issues involved, this diversity shall not be surprising.

Daily rhythms are not critical for determining the thermal limits of *Atta sexdens rubropilosa*. If sufficient numbers of individuals are available, assessments like ours may support the idea that testing at various times of the day will not increase the variance of the results. However, this trend is unlikely to be general because circadian clocks play a role in the expression of heat shock proteins [76] and because daily rhythms are a factor in the critical temperatures of other species, for example *Rana clamitans* [50]. If cyclic components of critical temperatures are more important in some models than in others, the only options are to make preliminary tests that verify this possibility or to take a conservative approach that aims to use measures according to ecologically relevant criteria, e.g., the time of day at which the maximum exposure to warm temperatures is likely to occur.

Protocols similar to those proposed in this paper would be useful to detect or rule out manipulation effects (independently of physiological mechanisms) and to better understand size-related effects on tests aiming to assess upper critical temperatures. In addition, these considerations may help authors interested in building upon published literature, for common ground is necessary [70]. The best data in this context would be collected according to the same standard protocols. However, if this is not possible, comparisons will require careful reading of the methods in each paper and the construction of a detailed dataset that includes the heating rate, time of day, season and actual geographic origin of the individuals included (not necessarily the native range). Special attention needs to be given to body mass and heating rates. Body mass needs to be considered for it may reflect both experimental artifacts and true physiological traits. Heating rates are likely to vary among samples, so that the interaction between body mass and heating rate needs to be explored within the limits of the data. In addition, because daily rhythms may be more pronounced in some species than in others, no general suggestions can be anticipated, and experimental controls for the time of day should be used. Finally, the criteria for endpoints and for pos-tests behavioral observation of experiments may generate different results.

## Materials and Methods

### Colonies

We used one laboratory colony of *Atta sexdens rubropulosa* containing approximately 25,000 individuals. We opted to use one colony because the physiological and methodological correlates of critical temperatures could be better analyzed when minimizing genetic variation in the sample. This colony was kept under natural photoperiod (from 10 h31 min D: 13 h29 min L to 11 h14 D: 12 h46 L) and at room temperature which varied throughout the experiment from 22°C to 27°C. It was regularly fed with leaves of *Acalipha* sp. *ad libitum*. The presence of immature forms was frequently monitored as the best possible indication of colony and queen health. At the time of the experiments, the colony was approximately 4 years old, appeared vigorous and displayed pots full of fungus and intense foraging activity. In addition, one mature natural colony was used to test for daily rhythms. This colony was located on the campus of the University of São Paulo. It is probable that this natural colony contained over 1,000,000 individuals.

### Critical Thermal Maximum

Our equipment was designed by Sable Systems (Las Vegas, USA) and consisted of a hotplate pelt with a programmable heating rate controlled by a computer interface. The temperature was monitored by two channels that measured the temperature independently and were simultaneously connected to a TC2000 Thermocouple Meter (Sable Systems). Because our general approach involved several hundred tests, we did not use of temperature sensors in the bodies of the ants. The system permitted ten ants to be tested simultaneously. Two ants were tested in each of five separate containers. We used only ants cutting or carrying leaves, regardless of body size. The temperature was increased at various rates (see Heating Rates). During the experiments, the ants were observed continuously and in the same order. Individual ants were rapidly turned upside down. The experiment ended when an individual could no longer return to the normal position within 5 seconds. The temperature was then recorded, and the ant was immediately placed in a small container at 25°C for recovery. Data points were only considered valid if an ant displayed normal activity two hours after a CTMax test. The ant was then weighed on a precision balance.

### Heating Rates

The equipment include a temperature controller allowing to control for heating rates and was calibrated to define the actual rate of warming of aluminum containers (6.2 cm in diameter and 2.4 cm deep) used to measure the CTMax of an ant. We measured the CTMax of 409 ants tested at 12 heating rates (Table 1).

### Daily Cycles

We used an adult colony of *Atta laevigata*. The mature colony exhibited vigorous trails and thousands of foraging ants. This is a phylogenetic related species to *Atta sexdens rubropilosa* and occupies a very similar niche [77]. At the time the experiments were carried out mature natural colonies of *Atta sexdens robropilosa* were unavailable on campus, and proximity to the laboratory was essential to minimize the time lag between capture and experiments. We preferred a natural colony for these tests to ensure the daily rhythms occurring in the field without interference from artificial lighting, feeding or maintenance schedules and to provide a natural temperature cycle. In this experiment, the heating rate used was always 1°C each 2 minutes. The experiment was conducted between 28/10/2010 and 12/01/2011 only on days without rain (these ants do not forage on rainy days) when ants were available (ants do not leave the nest on very hot days, e.g., >30°C). The ants were collected at the following times of day: 0:30, 3:30, 6:30, 9:30, 12:30, 15:30, 18:30 and 21:30. Three tests with 10 ants each were made at each time of day and on different days with this approach the time-lag between capture and experiment was always shorter than 10 min. In all, 232 ants were tested. We collected only ants returning to the nest with a piece of vegetation.

### Reproducibility of CTMax

In this experiment, we measured the CTMax of 76 ants. We followed the standard protocol with a heating rate of 0.5°C/min. The ants were returned to the colony after the test. All ants were marked on the head with a *PenTouch* pen (Sakura Color roducts corp, Osaka, Japan) for identification as tested or not-tested. This system does not allow ant individual recognition, but minimizes manipulation, time out of the colony and lost of chemical recognition marking, experimental priorities in these tests. After 24 hours, the CTMax of 41 marked ants was measured with the same protocol (0.5°C/min). After 24 additional hours (48 hours after the first test), 24 ants that had two marks (one from the first test and another from the second) were collected and again examined using the CTMax test protocol. No other protocol is viable because individual *Atta* deteriorate rapidly after 24 hours of isolation from the colony [42]. However, we investigated the possibility that replacing ants in the colony would enhance recovery. In this experiment, the CTMax values of 58 ants were measured following the standard protocol, but the second test (after the first CTMax exposure) occurred 144 hours later. We recovered 17 ants for this trial.

To control for experimental manipulation, we used 70 ants from the same colony. These ants were exposed to the protocol described above, but the hotplate remained off. In these tests, a fixed time (60 min) compatible with the actual assessment of the CTMax determined the end of the experiments. In 28 ants, we could apply this control twice before the actual readings of the CTMax (1°C every 2 minutes). To control for exposure to high but submaximal temperatures, we used 50 ants tested following the standard protocol with a heating rate of 0.5°C/min, but we used 35°C as an experimental endpoint. After 24 h, 27 ants that had been exposed to submaximal temperatures were subjected to normal CTMax assessment. The body mass was also measured again.

Following Berthou [78] we performed an ANCOVA in which heterogeneity of variance among treatments was controlled using logarithmic transformation. Due to the non normality of the obtained data, the reproducibility experiment was analyzed using permutation ANCOVA, following Manly's permutation method [79]. All analyses where done using R software (version 2.0.1)
